# 

**Published:** 1882-06

**Authors:** 


### CONTENTS.

### COMMUNICATIONS.

Chemistry in the Practice of Dentistry.................................... 261
Osseous Structure of the Teeth........................................ 268
The Management of Deciduous Teeth.................................. 276
The Relative Value of the Cuspidati.................................... 282
Relation of the Older Practitioner to the Younger........................ 287
Some Hints on the Preparation and Mounting of Microscopic Objects. 289
Aristocracy of Professions............................................... 297
Address.................................................................. 300
Vulcanizers and Vulcanizing............................................. 303

### CORRESPONDENCE.

From F. S. C., Edwards, Mississippi...................................... 305
Wooden Toothpicks....................................................... 306
American Dental Association............................................. 307

### EDITORIAL.

A Manual of Dental Anatomy, Human and Comparative.................. 308
Resorcine and its Employment in Therapeutics........................... 309
Indiana State Dental Association......................................... 310
Missouri State Dental Association........................................ 311
Kentucky State Dental Association........................................ 311
Changes.................................................................. 312
Manual of Dental Surgery and Pathology................................. 313
Arrangements for the Meeting of the American Dental Association... 314

